# The Relationship Between Paternal and Maternal Depression During the Perinatal Period: A Systematic Review and Meta-Analysis

**DOI:** 10.3389/fpsyt.2020.563287

**Published:** 2020-10-29

**Authors:** Freya Thiel, Merle-Marie Pittelkow, Hans-Ulrich Wittchen, Susan Garthus-Niegel

**Affiliations:** ^1^Department of Medicine, Faculty of Human Sciences, Medical School Hamburg, Hamburg, Germany; ^2^Faculty of Medicine, Institute and Policlinic of Occupational and Social Medicine, Technical University of Dresden, Dresden, Germany; ^3^Department of Psychometrics and Statistics, Faculty of Behavioural and Social Sciences, University of Groningen, Groningen, Netherlands; ^4^Department of Psychiatry and Psychotherapy, Ludwig-Maximilians-University Munich, Munich, Germany; ^5^Department of Child Health and Development, Norwegian Institute of Public Health, Oslo, Norway

**Keywords:** depression, paternal, maternal, parental, perinatal, pregnancy, postnatal, childbirth

## Abstract

**Background:** Meta-analyses suggest an increased prevalence of paternal depression during the perinatal period of around 10%. The relationship between paternal and maternal symptoms, however, has received little attention.

**Objective:** To determine pooled estimates pertaining to the relationship between paternal and maternal depression during the perinatal period according to the Preferred Reporting Items for Systematic Reviews and Meta-Analyses statement.

**Data sources:** Studies reporting on the relationship between depression in fathers and mothers between the first trimester and the first year following childbirth were identified using PubMed, PsycINFO, and EMBASE for the period between November 2009 and February 2020.

**Study selection:** A total of 28 primary, empirical studies published in English or German, reporting effect estimates for the relationship of depression in mother–father/partner dyads, involving 11,593 couples, were included. Ten studies included multiple assessments, resulting in 64 extracted effects.

**Analysis:** Information on correlations and odds ratios were extracted. Four random-effects analyses were conducted for the pooled association between paternal and maternal depression: (a) during the prenatal and (b) during the postnatal period, as well as for the prospective relationships between (c) paternal depression and maternal depression at a later timepoint, and (d) vice versa. Models were specified as restricted maximum-likelihood estimation. Heterogeneity was assessed using *H*^2^ and *I*^2^. Funnel plots, the Egger method, and the trim-and-fill test were used to assess publication bias. Sensitivity analyses with and without studies for which we approximated *r* were conducted.

**Data synthesis:** With substantial heterogeneity, positive associations were found between paternal and maternal depression (a) during pregnancy (*r* = 0.238), (b) in the postnatal period (*r* = 0.279), as well as for the prospective relationship between (c) paternal and later maternal depression (*r* = 0.192), and (d) maternal and later paternal depression (*r* = 0.208).

**Conclusion:** Paternal depression showed positive correlations with maternal depression across the perinatal period. Given notable methodological and cultural heterogeneity and limitations of individual studies, it was not possible to further identify determining or moderating factors. Increasing evidence for implications of parental depression for child development warrants further scientific attention.

## Introduction

A large body of research has established the adverse implications of maternal symptoms of depression during pregnancy and following birth for mothers, the family system, and child development. In fact, with incidence rates between 10 and 30%, maternal postnatal depression has been documented as the most frequent complication of childbirth (1–6). Although less empirical attention has been devoted to paternal depression, there is evidence that fathers are at an increased risk of depression during pregnancy and the postnatal period as well (7–11).

Meta-analyses of studies on prevalence suggest that around 10% of men experience depression during the perinatal period (10, 12). During pregnancy, paternal prenatal depression may peak in the third trimester, with prevalence between 9 and 12% (10, 12). Following birth, paternal postnatal depression may peak at 3 to 6 months, with rates of up to 26% (10). Similar to findings on the adverse effects of maternal psychopathology on mothers' health, birth complications, and child short- and long-term development (13–16), several studies have reported associations between paternal depression and negative child outcomes (17–19).

Given that one can assume particular negative effects if both parents are affected, it is remarkable that little is known about the frequency, nature, and the effects of the association of paternal and maternal depression in the perinatal period. Depression affects not only the individual, but also the wider social context (20). Within couples, shared environmental and interpersonal (i.e., relationship) stressors are likely to affect both parents and may ultimately contribute to the development of depressive symptoms. This impact, however, may be bidirectional in nature. For instance, compared to families without depression, families with a depressed mother report a higher number of stressors (21), as well as higher stress in various domains, such as work, relationships, or children (22). In the family context, marital dissatisfaction or conflict represents stressors of particular importance because they may affect all family members (23). Not surprisingly, marital conflict is associated with depression (24, 25), and among depressed women, rates of marital conflict and divorce are elevated (26, 27). Further, marital dissatisfaction explains 18% of variance in wives' and 14% of variance in husbands' depressive symptoms (28). Thus, couples seem to be particularly vulnerable to co-occurring depressive symptoms. This vulnerability may be augmented by additional stressors related to pregnancy and the transition to parenthood.

To date, only one meta-analysis has evaluated the relationship between paternal and maternal symptoms of depression in the perinatal period (10). Based on 43 studies published between 1980 and 2009, Paulson and Bazemore (10) reported a positive, yet moderate correlation between paternal and maternal depression. Although this initial meta-analysis has been updated by Cameron and colleagues (12) with regard to prevalence of paternal depression, a more recent meta-analytic synthesis pertaining to the association between paternal and maternal depression in the perinatal period is lacking. Since the completion of the last meta-analysis in 2009, further studies reporting on this association have been published, rendering an additional systematic review and meta-analysis necessary.

The current study therefore provides a systematic review and meta-analysis of the association between parental perinatal depression in expectant and new fathers and mothers, taking into consideration the conditional effect of assessment time, with a distinguishing focus on prenatal and postnatal as well as prospective effects.

## Methods

### Protocol

The study was designed and written according to the Preferred Reporting Items for Systematic Reviews and Meta-Analyses statement.

### Eligibility Criteria

For inclusion in this meta-analysis, we considered primary, empirical studies published in English or German, using quantitative analysis with measures of paternal and maternal depression. We included studies reporting effect estimates for the relationship of symptoms of depression in mother–father/partner dyads. This comprised both observational studies (including cohort, cross-sectional, and clinical studies) and experimental studies. If a study published multiple times on an overlapping sample, the most recently written article was included. If a study reported multiple effect estimates for different timepoints, all measurements were extracted. Conference abstracts, case studies, dissertations/theses with a peer-reviewed published version, and studies with nonhuman subjects were excluded.

### Information Sources and Literature Search

Electronic PsycINFO, MEDLINE, and EMBASE searches were conducted from November 2009 to February 17, 2020, using the search string “*depression* AND *TI* (*paternal* OR *father* OR *partner*) AND *TI* (*perinatal* OR *prenatal* OR *postnatal* OR *postpartum* OR *peripartum* OR *pregnancy* OR *childbirth*)” for both PsychINFO and MEDLINE. For EMBASE, the string was adapted to (“depression”/exp OR depression) AND (paternal OR “father”/exp OR father OR “partner”/exp OR partner) AND (“ti”/exp OR ti) AND (perinatal OR “prenatal”/exp OR prenatal OR postnatal OR “postpartum”/exp OR postpartum OR peripartum OR “pregnancy”/exp OR pregnancy OR “childbirth”/exp OR childbirth).

### Study Selection

Screening was conducted in Excel. After removing duplicate articles, titles and abstracts were screened. Studies reporting an association between paternal and maternal depression in the perinatal period were included. This resulted in the exclusion of several studies that did not include parent couples or only examined paternal and maternal depression separately from one another.

### Summary Measure

If possible, Pearson and Spearman correlation coefficients (*r*) were extracted. Alternatively, we calculated approximations of the correlation coefficient for studies reporting odds ratios (ORs), we followed Digby (29) and used the following tetrachoric approximation: $r=\frac{\left(OR^{3/4}-1\right)}{\left(OR^{3/4}+1\right)}$ (30).

### Data Collection Process

We developed a data extraction sheet in Excel and pilot tested it in a random sample of 10 included studies and refined it accordingly.

### Methods of Analysis

Analyses were conducted in R Studio 1.1.456 using the *metaphor* package (31). Four separate random-effects meta-analyses were conducted. First, the pooled association between paternal and maternal depression during pregnancy was estimated. Second, the pooled association between paternal and maternal depression in the postnatal period was estimated. Lastly, we additionally pooled effects concerning the prospective relationships between perinatal paternal depression at one timepoint and maternal depression at a later timepoint and vice versa. Models were specified as restricted maximum-likelihood estimation providing an approximately unbiased and efficient estimator of heterogeneity (32). Heterogeneity was assessed using *H*^2^, an estimate of between-study heterogeneity, and *I*^2^, an estimate of the total variance explained by heterogeneity.

#### Risk of Bias Across Studies

Visual inspection of funnel plots, Egger et al. (33) regression test of funnel plot asymmetry and the trim-and-fill test (34) were used to assess evidence for publication bias.

#### Sensitivity Analyses

We performed sensitivity analyses with and without studies for which we approximated *r* to inspect the effect our calculations had on the pooled estimates.

## Results

### Study Selection

**Figure 1:**
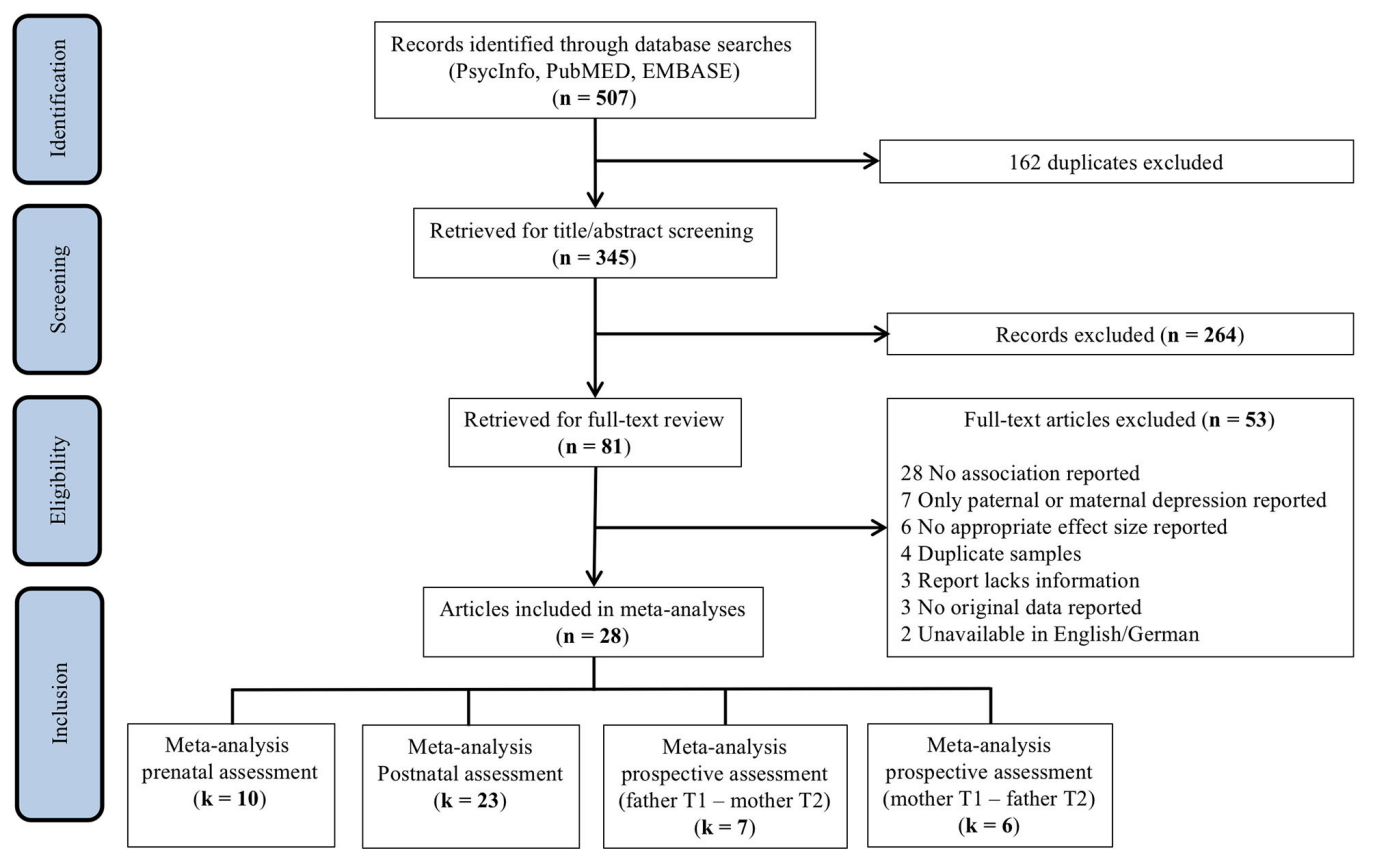
Study selection for inclusion in meta-analyses.

The search of PsycINFO, MEDLINE, and EMBASE provided a total of 507 articles. After removing duplicates, the title and abstract of 345 studies were screened. After title/abstract screening, 264 studies were discarded as they did not meet the prespecified inclusion criteria. Thus, 81 studies were retrieved for full-text screening. Of these, 53 studies were excluded as they did not report associations between paternal and maternal depression, only reported on either paternal or maternal depression, included duplicate samples, lacked sufficient information, reported no original data, or were unavailable in English or German. In addition, two studies examined paternal depressive symptoms as a predictor of maternal depression in multivariate models (35, 36), and two reported concordance between parental depression trajectories across the perinatal period through cross-tabs (37, 38). Although these studies will be discussed below, they were not included in the current meta-analysis because reported estimates could not be statistically integrated. An overview of the study selection process can be found in Figure 1.

The screening process resulted in a total of 28 articles. Of these studies, 18 reported a single observation, whereas 10 included multiple assessments at different timepoints, resulting in a total of 64 extracted effects. Inclusion of multiple effect sizes from a single sample compromises the independence assumption of meta-analysis (39). In order to minimize potential attrition biases, primary analyses include the earliest reported estimate, which is generally based on a larger preattrition sample size. Selection yielded a total of 40 effects, which were classified as assessing either the association between paternal and maternal depression (a) during pregnancy (prenatal assessment), (b) during the postnatal period (postnatal assessment), or (c) the prospective association of perinatal paternal depression at one timepoint on maternal depression at a later timepoint. The meta-analyses pertaining to prenatal, postnatal, and prospective assessments included *k* = 10 (40–49), *k* = 23 (42, 45, 47–67), and *k* = 7 (42, 47, 50, 52, 55, 60, 61) effects, respectively (Figure 1).

### Study Characteristics

The included studies varied in design comprising cross-sectional (*n* = 11) and longitudinal (*n* = 17) designs. For characteristics of the 28 studies included in the meta-analyses (31–58), refer to Tables 1A–D. Although studies originated in 16 countries, half of them came from Italy (*n* = 7 studies) and the United States (*n* = 7 studies). Whereas, the majority of studies (*n* = 27) used self-report scales to assess parental depressive symptoms [*n* = 22: Edinburgh Postnatal Depression Scale (EPDS), *n* = 2: Center for Epidemiologic Studies Depression Scale (CES-D), *n* = 1: Beck Depression Inventory (BDI), *n* = 1: Hospital Anxiety and Depression Scale (HADS), *n* = 1: Brief Symptom Inventory (BSI)], one study employed the Structured Clinical Interview for the *DSM*. Two studies reported associations of parental depression using both the EPDS and the CES-D (47, 65). As noted above, inclusion of multiple effect sizes from a single sample compromises the independence assumption of meta-analysis (39). Although CES-D estimates for these studies can be found in Tables 1A–D for additional information, only EPDS estimates were included in the primary analyses because this measure was implemented in the majority of the other studies, making the results more comparable.

**Table 1A:** Characteristics of studies included in meta-analysis for the association between paternal and maternal depression during the prenatal period.

**Source**	**Assessment time**	**Location**	**Depression measure**	**No. couples**	**Correlation paternal and maternal depression**
**Prenatal assessment (*****n*** **= 10)**					
Ahlqvist-Björkroth et al. (40)	GW 20	Finland	EPDS	147	0.21^*^
Brandão et al. (41)	Average GW 32	Portugal	HADS	320	0.27^*^
Cussino et al. (42)	Average GW 36	Italy	EPDS	63	0.24
El Marroun et al. (43)	Average GW 26	Netherlands	BSI	461	0.21^*^
Formica et al. (44)	GW 17–23	Italy	CES-D	40	0.13
Gürber et al. (45)	GW 20–25	Switzerland	EPDS	140	0.11
Nasreen et al. (46)	Third trimester	Malaysia	EPDS	583	3.95^a^^,^ ^*^
Paulson et al. (47)	GW 28	US	EPDS	78	0.27^*^
Stramrood et al. (48)	During pregnancy	Netherlands	BDI-II	85	0.17
Top et al. (49)	GW 37–40	Turkey	EPDS	92	0.09

*GW, gestational week; BDI, Beck Depression Inventory; BSI, Brief Symptom Inventory; CES-D, Center of Epidemiologic Studies Depression Scale; EPDS, Edinburgh Postnatal Depression Inventory; HADS, Hospital Anxiety Depression Scale*.

a *Odds ratio*.

* *p < 0.05*.

**Table 1B:** Characteristics of studies included in meta-analysis for the association between paternal and maternal depression during the postnatal period.

**Source**	**Assessment time**	**Location**	**Depression measure**	**No. couples**	**Correlation paternal and maternal depression**
**Postnatal assessment (*****n*** **= 23)**					
Anding et al. (54)	Within 2 weeks	Germany	EPDS	276	0.30^*^
Ayinde and Lasebikan (55)	6 weeks	Nigeria	SCID-I	331	0.06
Cheng et al. (56)	9 months	US	CES-D	5350	0.24^*^
Cussino et al. (42)	Average 12 weeks	Italy	EPDS	63	0.38^*^
Epifanio et al. (57)	1 month	Italy	EPDS	53	−0.11
Gürber et al. (45)	1 month	Switzerland	EPDS	140	0.39^*^
Gao et al. (58)	6–8 weeks	China	EPDS	130	0.37^*^
Goyal et al. (59)	48 h	India	EPDS	479	0.95^*^
Gutierrez-Galve et al. (60)	8 weeks	UK	EPDS	3176	0.27^*^
	8 months^a^				0.28^*^^,^^*a*^
Iles et al. (61)	6 weeks	UK	EPDS	212	0.30^*^
	3 months^a^				0.19^*^^,^ ^*a*^
Kerstis et al. (62)	3 months	Sweden	EPDS	249	0.29^*^
Massoudi et al. (63)	3 months	Sweden	EPDS	858	0.23^*^
Nishigori et al. (64)	1 month	Japan	EPDS	1023	1.53^b^
Nishimura et al. (66)	4 months	Japan	EPDS	807	1.9^b^^,^ ^*^
Nishimura et al. (65)	1 month	Japan	EPDS	129	0.10
			CES-D^a^	129	0.16^a^
Paulson et al. (47)	1 month	US	EPDS	78	0.05
	3 months^a^				0.28^*^^,^ ^*a*^
	6 months^a^				0.42^*^
	1 month^a^		CES-D^a^		0.28^*^^,^ ^*a*^
	3 months^a^				0.26^*^^,^ ^*a*^
	6 months^a^				0.31^*^^,^ ^*a*^
Roubinov et al. (67)	15 weeks	US	EPDS	92	0.05
Saxbe et al. (50)	1 month	US	EPDS	711	0.25^*^
	6 months^a^				0.27^*^^,^ ^*a*^
	12 months^a^				0.26^*^^,^ ^*a*^
Smith et al. (51)	3 months	UK	EPDS	705	0.22^*^
Stramrood et al. (48)	6 weeks	Netherlands	BDI-II	85	0.59^*^
Top et al. (49)	4–6 weeks	Turkey	EPDS	92	0.19
Vismara et al. (52)	3 months	Italy	EPDS	181	0.54^*^
	6 months^a^				0.44^*^^,^ ^*a*^
Wynter et al. (53)	6 months	Australia	EPDS	172	0.18^*^

*GW, gestational week; BDI, Beck Depression Inventory; CES-D, Center of Epidemiologic Studies Depression Scale; EPDS, Edinburgh Postnatal Depression Inventory; SCID-I, Structured Clinical Interview for the DSM*.

a *Not included in the analysis*.

b *Odds ratio*.

* *p < 0.05*.

**Table 1C:** Characteristics of studies included in meta-analysis for the prospective association between paternal depression at one timepoint and maternal depression at a later timepoint.

**Source**	**Assessment time**	**Location**	**Depression measure**	**No. couples**	**Correlation paternal and maternal depression**
**Prospective assessment T1 father–T2 mother (*****n*** **= 7)**					
Ayinde and Lasebikan (55)	At birth−6 weeks PN	Nigeria	SCID-I	331	0.01
Cussino et al. (42)	Average GW 36–12 weeks PN	Italy	EPDS	63	0.34^*^
Gutierrez-Galve et al. (60)	8 weeks−8 months PN	UK	EPDS	3176	0.21^*^
Iles et al. (61)	6 weeks−3 months PN	UK	EPDS	212	0.28^*^
Paulson et al. (47)	GW 28–1 month PN	US	EPDS	78	0.15
	GW 28–3 months PN^a^				0.28^*^
	GW 28–6 months PN^a^				0.28^*^
	1–3 months PN^a^				0.29^*^
	1–6 months PN^a^				0.25^*^
	3–6 months PN^a^				0.33^*^
	GW 28–1 month PN^a^		CES-D^a^	78	0.27^*^
	GW 28–3 months PN^a^				0.24^*^
	GW 28–6 months PN^a^				0.32^*^
	1–3 months PN^a^				0.28^*^
	1–6 months PN^a^				0.22^*^
	3–6 months PN^a^				0.27^*^
Saxbe et al. (50)	1–6 months PN	US	EPDS	711	0.19**
	1–12 months PN^a^				0.20^*^
	6–12 months PN^a^				0.18^*^
Vismara et al. (52)	3–6 months PN	Italy	EPDS	181	0.23^*^

*GW, gestational week; PN, postnatal; CES-D, Center of Epidemiologic Studies Depression Scale; EPDS, Edinburgh Postnatal Depression Inventory; SCID-I, Structured Clinical Interview for the DSM*.

a *Not included in the analysis*.

* *p < 0.05*.

**Table 1D:** Characteristics of studies included in meta-analysis for the prospective association between maternal depression at one timepoint and paternal depression at a later timepoint.

**Source**	**Assessment time**	**Location**	**Depression measure**	**No. couples**	**Correlation paternal and maternal depression**
**Prospective assessment T1 father–T2 mother (*****n*** **= 6)**					
Cussino et al. (42)	Average GW 36–12 weeks PN	Italy	EPDS	63	0.35^*^
Gutierrez-Galve et al. (60)	8 weeks−8 months PN	UK	EPDS	3176	0.22^*^
Iles et al. (61)	6 weeks−3 months PN	UK	EPDS	212	0.18^*^
Paulson et al. (47)	GW 28–1 month PN	US	EPDS	78	0.11
	GW 28–3 months PN^a^				0.17
	GW 28–6 months PN^a^				0.14
	1–3 months PN^a^				0.10
	1–6 months PN^a^				−0.04
	3–6 months PN^a^				0.27^*^
	GW 28–1 month PN^a^		CES-D^a^	78	0.28^*^
	GW 28–3 months PN^a^				0.21
	GW 28–6 months PN^a^				0.20
	1–3 months PN^a^				0.13
	1–6 months PN^a^				0.17
	3–6 months PN^a^				0.07
Saxbe et al. (50)	1–6 months PN	US	EPDS	711	0.14^*^
	1–12 months PN^a^				0.21^*^
	6–12 months PN^a^				0.24^*^
Vismara et al. (52)	3–6 months PN	Italy	EPDS	181	0.27^*^

*GW, gestational week; PN, postnatal; CES-D, Center of Epidemiologic Studies Depression Scale; EPDS, Edinburgh Postnatal Depression Inventory*.

a *Not included in the analysis*.

* *p < 0.05*.

Ten studies (36%) were based on community- or population-based samples within birth cohort studies, three studies (11%) recruited from maternity or postpartum units, and 14 studies recruited from parenting/prenatal classes and other health services (50%). Of the 28 studies, 19 reported whether couples were married/cohabiting—in nine studies, all couples were married/cohabiting; percentages of married/cohabiting couples in the remaining 10 studies varied from 49% (67) to 99% (51). Similarly, 21 studies reported primiparity proportions within the sample. Four studies included only primiparous parents, and the proportion of primiparous couples in the other studies ranged from 36% (46) to 87% (42). One study focused on low-income couples with Mexican–American fathers (67). Response rates were reported in 19 studies and ranged from 26 to 97.5%, with a median of 65% (first quartile = 50%, third quartile = 90%). Sample sizes varied widely across studies (n = 40–5,350 couples), with a median of 231 participants (first quartile = 111, third quartile = 644). Overall, using sample sizes across the 28 studies, a total of 11,593 couples, that is, 23,186 participants, are represented in this meta-analysis.

### Data Synthesis

#### Association of Paternal and Maternal Depression During the Prenatal Period

##### Central tendency and variability

**Figure 2:**
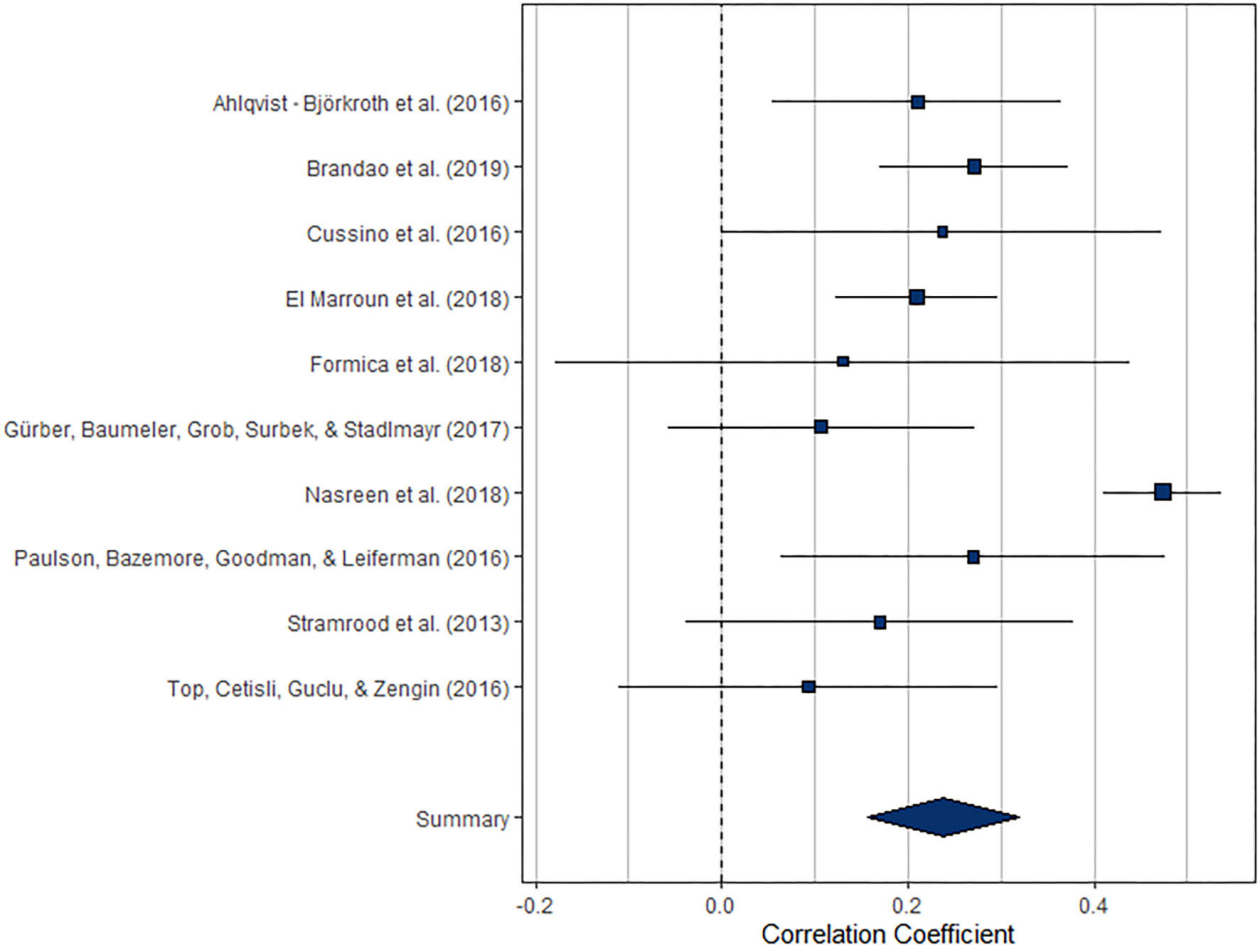
Forest plot of the analysis of the association between paternal and maternal depression during the prenatal period. Weighted effect size and 95% CI are presented on the right.

Overall, the pooled association between paternal and maternal depression during pregnancy (*k* = 10) was statistically significant, *r* = 0.238 [95% confidence interval (CI) [0.157, 0.320], *z* = 5.71, *p* < 0.0001]. For a graphical representation, refer to Figure 2. All associations reported were positive without exemption.

The index of heterogeneity between the studies *H*^2^ = 3.25 (95% CI [1.58, 8.86]) was significant, *Q*(9) = 47.0, *p* < 0.0001, suggesting that the observed variability in the effects is larger than would be expected based on the sampling variance *I*^2^ = 69.25% (95% CI [36.80, 88.71]).

##### Risk of bias across studies

**Figure 3:**
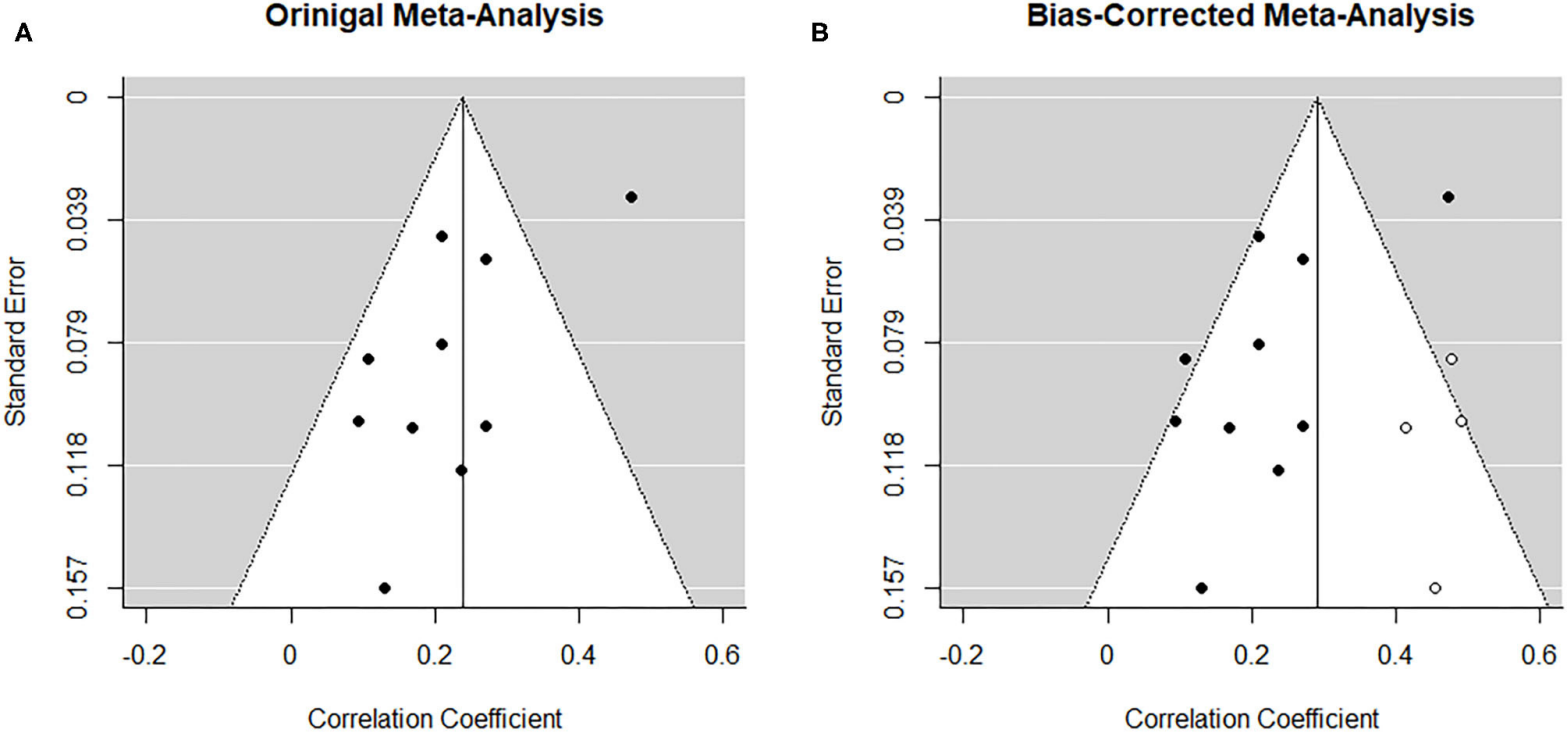
Funnel plots before **(A)** and after **(B)** applying trim-and-fill method corresponding to the meta-analysis on the association between paternal and maternal depression during the prenatal period. The open dots indicate imputed studies.

From visual inspection, the funnel plot (Figure 3A) appears asymmetrical. Based on the large heterogeneity observed between studies, a random-random effects trim and fill model was implemented to check for the presence of publication bias (34) (Figure 3B). The overall pooled estimate increased to *r* = 0.291 (95% CI [0.216, 0.366], *z* = 7.57, *p* < 0.001) after imputing four possible missing studies on the bottom right.

##### Sensitivity analysis

To control for the effect that the tetrachoric association might have on the pooled estimate, we performed a sensitivity analysis, excluding the study reporting an OR. Although exclusion of one study (46) decreased the overall pooled estimate slightly, *r* = 0.208 (95% CI [0.159, 0.258]), it remained statistically significant (*z* = 8.23, *p* < 0.0001).

#### Association of Paternal and Maternal Depression During the Postnatal Period

##### Central tendency and variability

**Figure 4:**
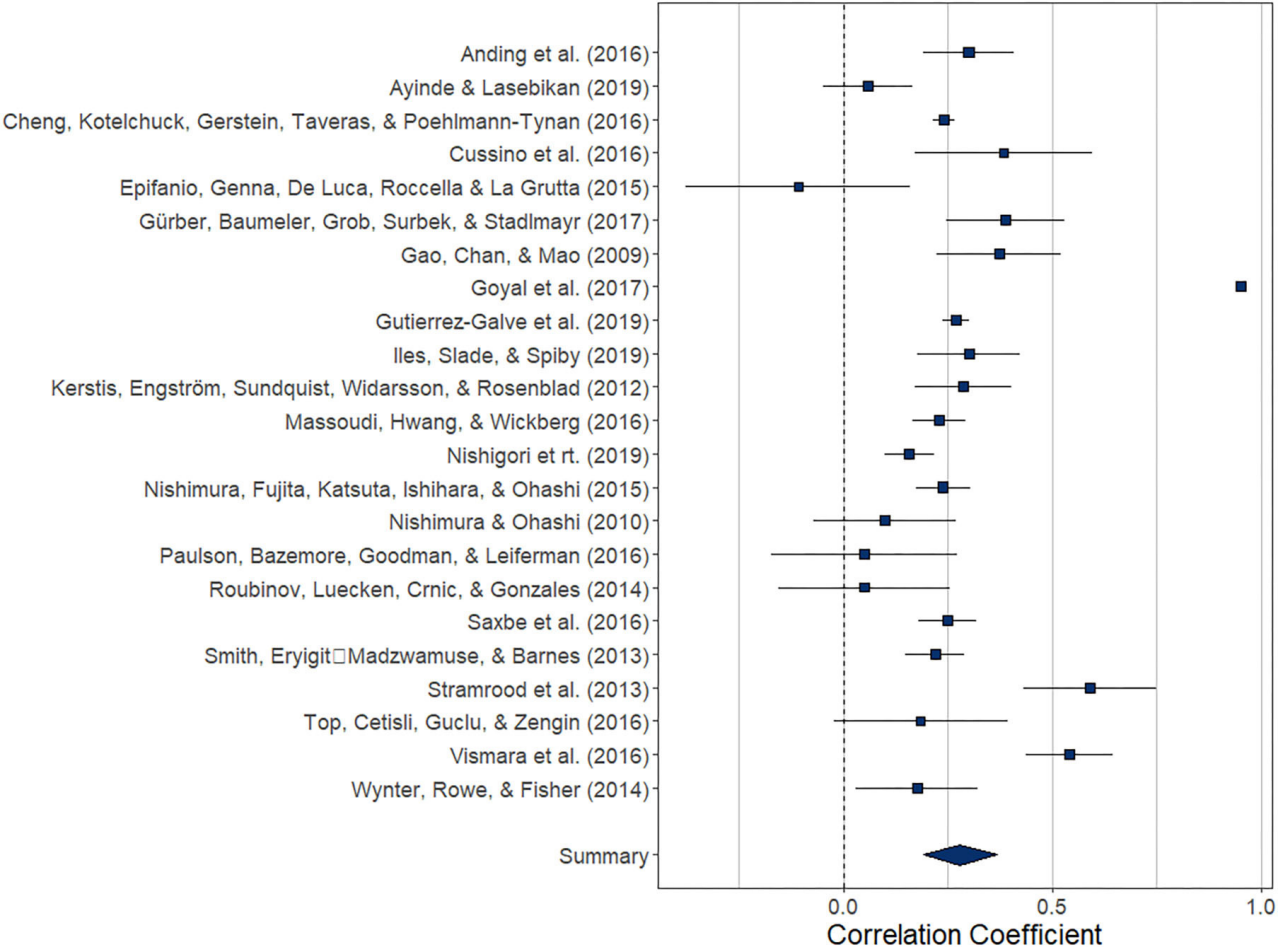
Forest plot of the analysis of the association between paternal and maternal depression during the postnatal period. Weighted effect size and 95% CI are presented on the right.

Overall, the pooled association between paternal and maternal depression during the postnatal period (*k* = 23) was statistically significant, *r* = 0.279 (95% CI [0.192, 0.367], *z* = 6.29, *p* < 0.0001). For a graphical representation, refer to Figure 4. Except for one study, all reported associations were positive.

The index of heterogeneity between the studies *H*^2^ = 54.56 (95% CI [32.14, 117.28) was significant, *Q*(22)= 6376.4, *p* < 0.0001, suggesting that the observed variability in the effects is larger than would be expected based on the sampling variance *I*^2^ = 98.17% (95% CI [96.89, 99.15]).

##### Risk of bias across studies

**Figure 5:**
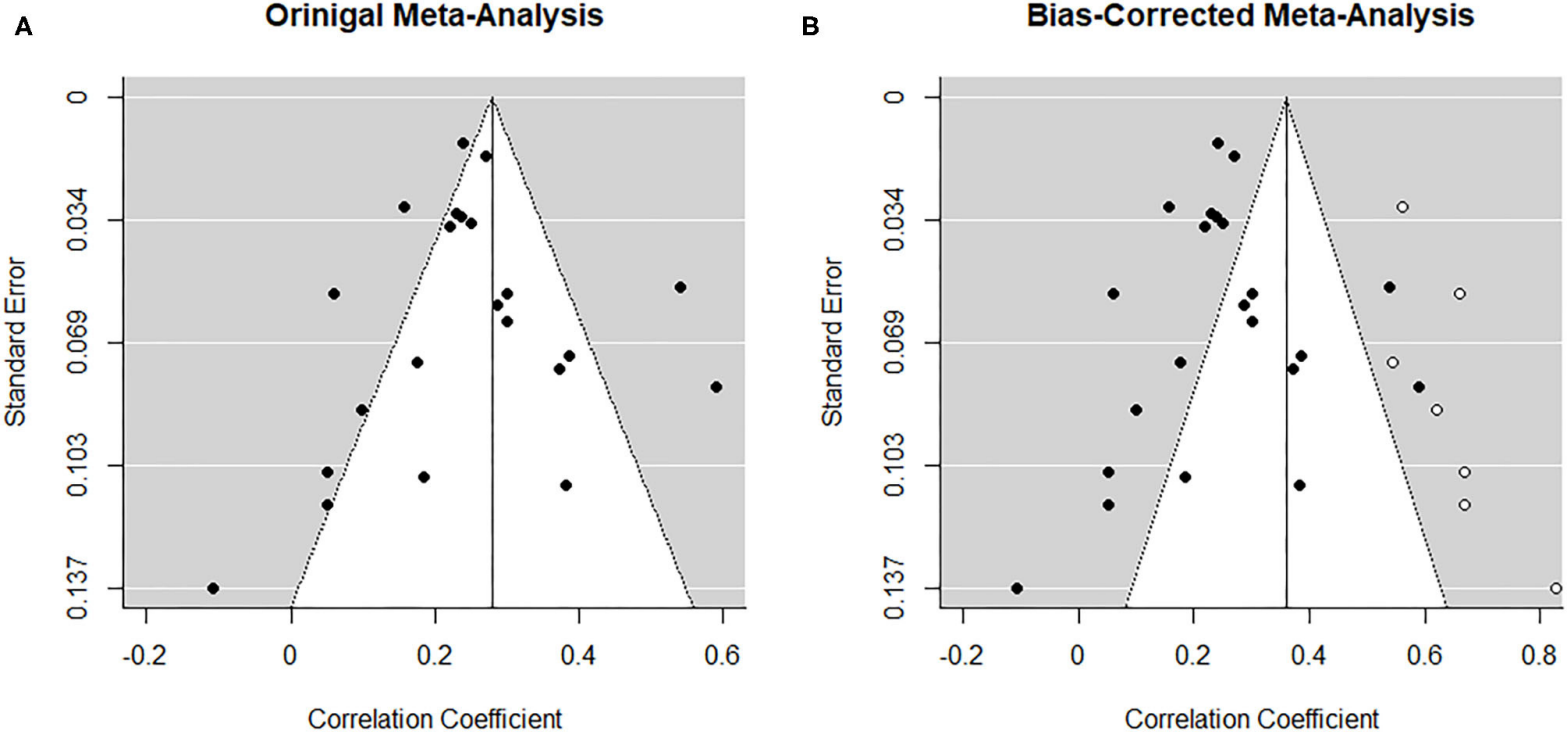
Funnel plots before **(A)** and after **(B)** applying trim-and-fill method corresponding to the association between paternal and maternal depression during the postnatal period. The open dots indicate imputed studies.

From visual inspection, the funnel plot (Figure 5A) appears symmetrical and thus does not point toward the influence of publication bias on the results. Nonetheless, based on the heterogeneity observed between studies, a random-effects trim-and-fill model was implemented to check for the presence of publication bias (34) (Figure 5B). The overall pooled estimate increased to *r* = 0.360 (95% CI [0.274, 0.446], *z* = 8.18, *p* < 0.001) after imputing seven possible missing studies on the right. Similarly, the regression test for funnel plot asymmetry (33) was statistically significant (*z* = −2.04, *p* = 0.04).

##### Sensitivity analysis

To control for the effect that the tetrachoric association might have on the pooled estimate, we performed a sensitivity analysis, excluding the two studies reporting an OR (64, 66). Moreover, based on influential case analysis, we excluded one study (59) reporting an unusually high correlation of *r* = 0.95. Although exclusion of the three effects decreased the pooled estimate slightly (*r* = 0.256 (95% CI [0.192, 0.319]), it remained significant (*z* = 7.96, *p* < 0.0001).

#### Prospective Association Between Paternal and Maternal Depression During the Perinatal Period

##### Central tendency and variability

**Figure 6:**
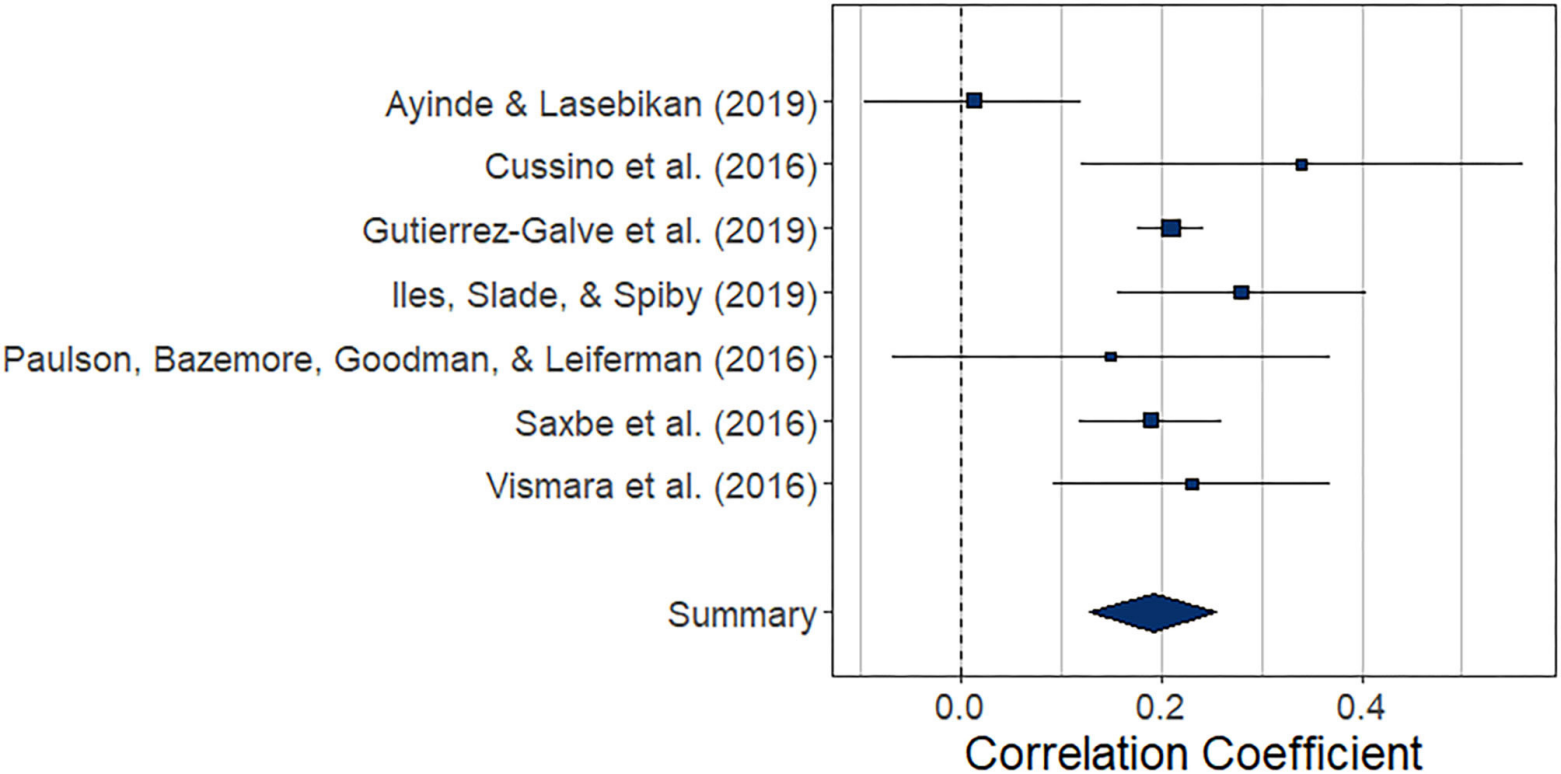
Forest plot of the analysis of the prospective association between paternal depression at one timepoint and maternal depression at a later timepoint during the perinatal period. Weighted effect size and 95% CI are presented on the right.

The meta-analysis regarding the prospective association between paternal depression at one point and maternal depression at a later timepoint during the perinatal period (*k* = 7) yielded statistically significant results, *r* = 0.192 (95% CI [0.129, 0.255], *z* = 5.97, *p* < 0.0001). For a graphical representation, refer to Figure 6. All prospective associations reported were positive without exemption. Note that this meta-analysis is based on a small set of studies, thus limiting the stability of estimated effects.

The index of heterogeneity between the studies *H*^2^ = 2.67 (95% CI [1.10, 21.69) was significant, *Q*(6)= 15.49, *p* = 0.02, suggesting that the observed variability in the effects is larger than would be expected based on the sampling variance *I*^2^ = 62.35% (95% CI [9.08, 95.39]).

##### Risk of bias across studies

**Figure 7:**
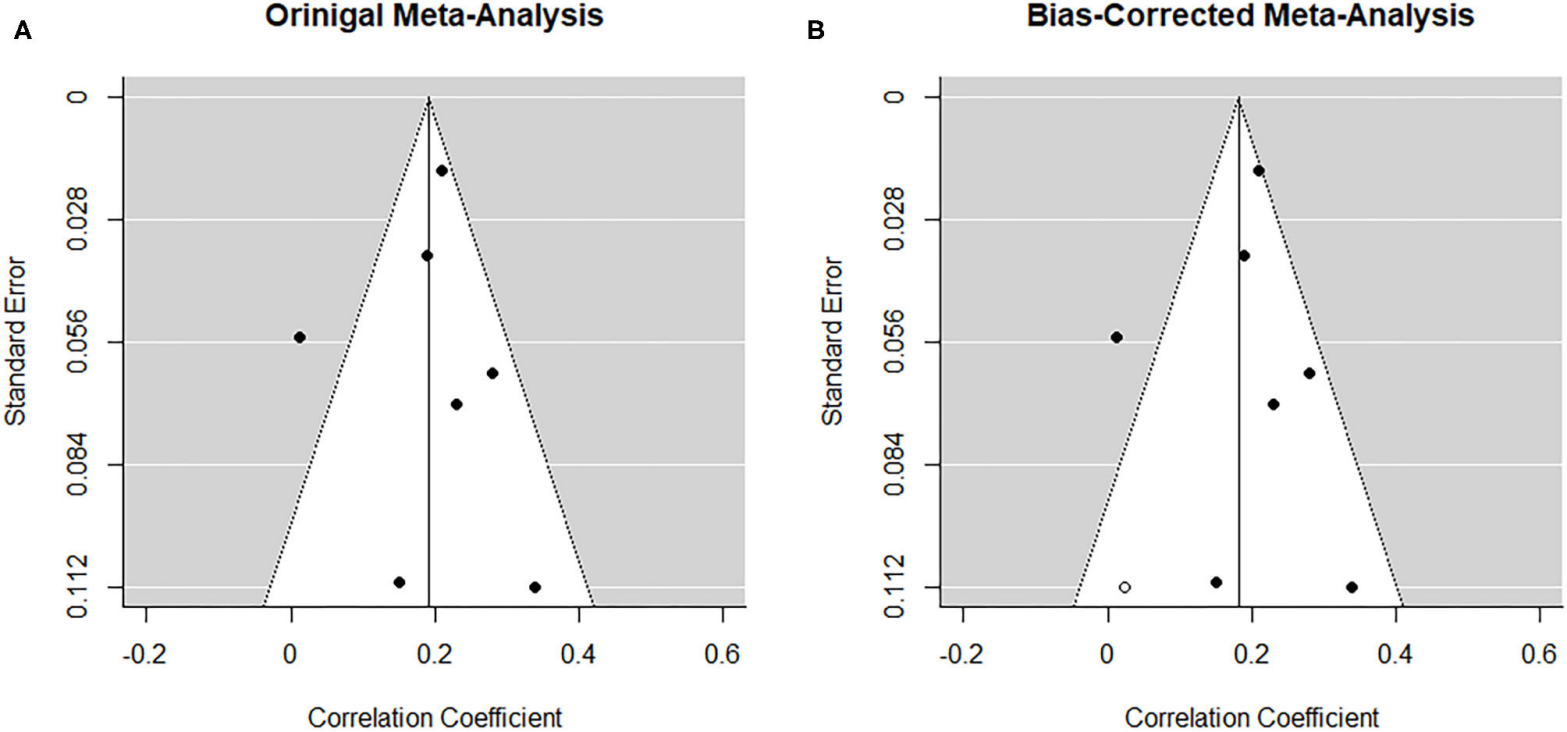
Funnel plots before **(A)** and after **(B)** applying trim-and-fill method corresponding to the prospective association between paternal depression at one timepoint and maternal depression at a later timepoint during the perinatal period. The open dots indicate imputed studies.

The low number of studies limits interpretability of the following procedures. From visual inspection, the funnel plot (Figure 7A) appears symmetrical. In line with the visual inspection, the regression test for funnel plot symmetry (33) was not statistically significant (*z* = 0.60, *p* = 0.548). The trim-and-fill procedure (34), however, suggests one missing study on the left side (Figure 7B). After imputing this value, the overall pooled estimate decreases to *r* = 0.182 (95% CI [0.12, 0.24], *z* = 5.67, *p* < 0.001).

#### Prospective Association Between Maternal and Paternal Depression During the Perinatal Period

##### Central tendency and variability

**Figure 8:**
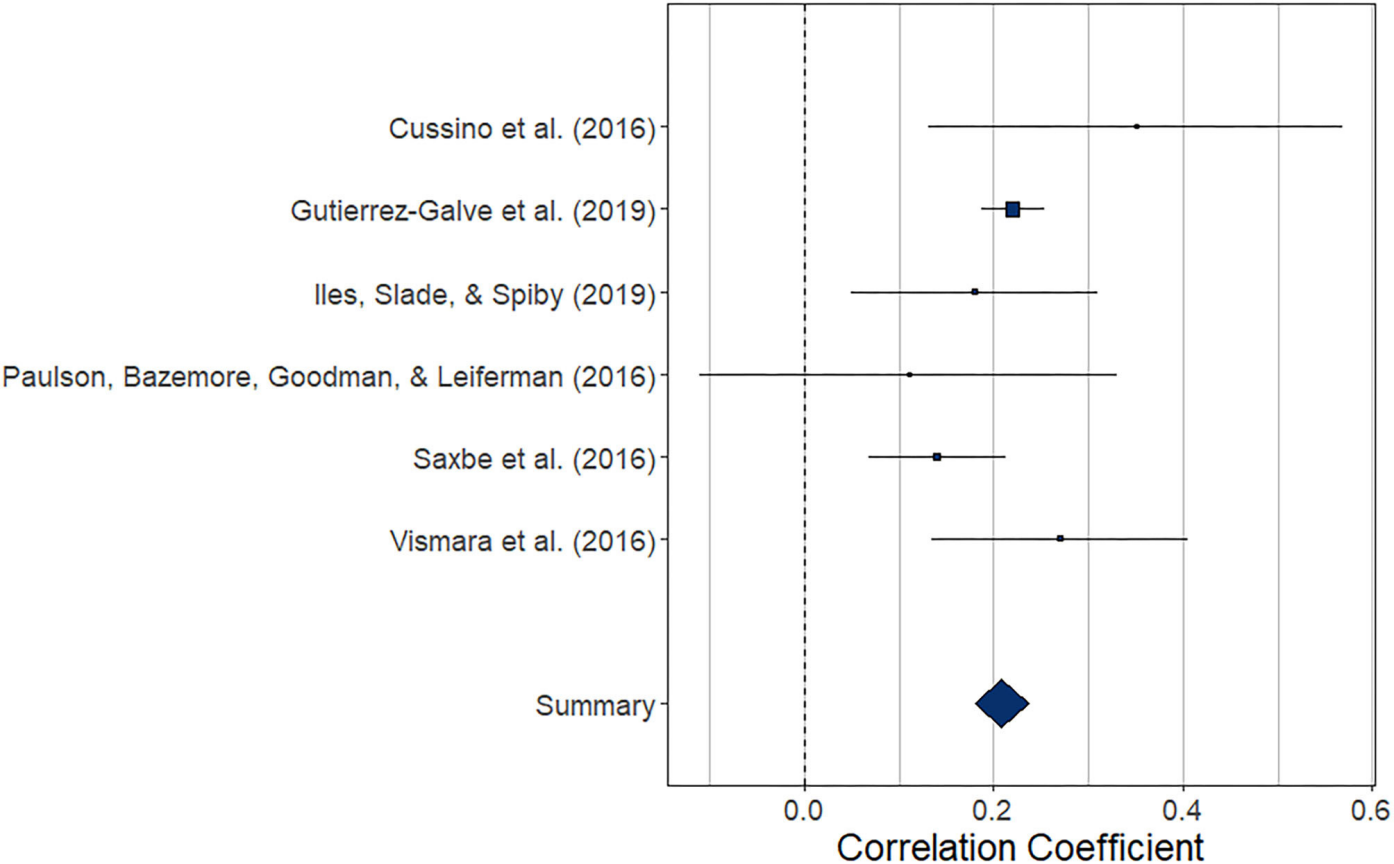
Forest plot of the analysis of the prospective association between maternal depression at one timepoint and paternal depression at a later timepoint during the perinatal period. Weighted effect size and 95% CI are presented on the right.

The meta-analysis regarding the prospective association between maternal depression at one point and paternal depression at a later timepoint during the perinatal period (*k* = 6) yielded statistically significant results, *r* = 0.208 (95% CI [0.180, 0.237], *z* = 14.48, *p* < 0.0001). For a graphical representation, refer to Figure 8. All but one of the prospective associations reported were positive. Note that again, this meta-analysis is based on a small set of studies, thus limiting the stability of estimated effects.

The index of heterogeneity between the studies *H*^2^ = 1.00 (95% CI [1.00, 17.13]) was not significant, *Q*(5)= 7.28, *p* = 0.20.

##### Risk of bias across studies

**Figure 9:**
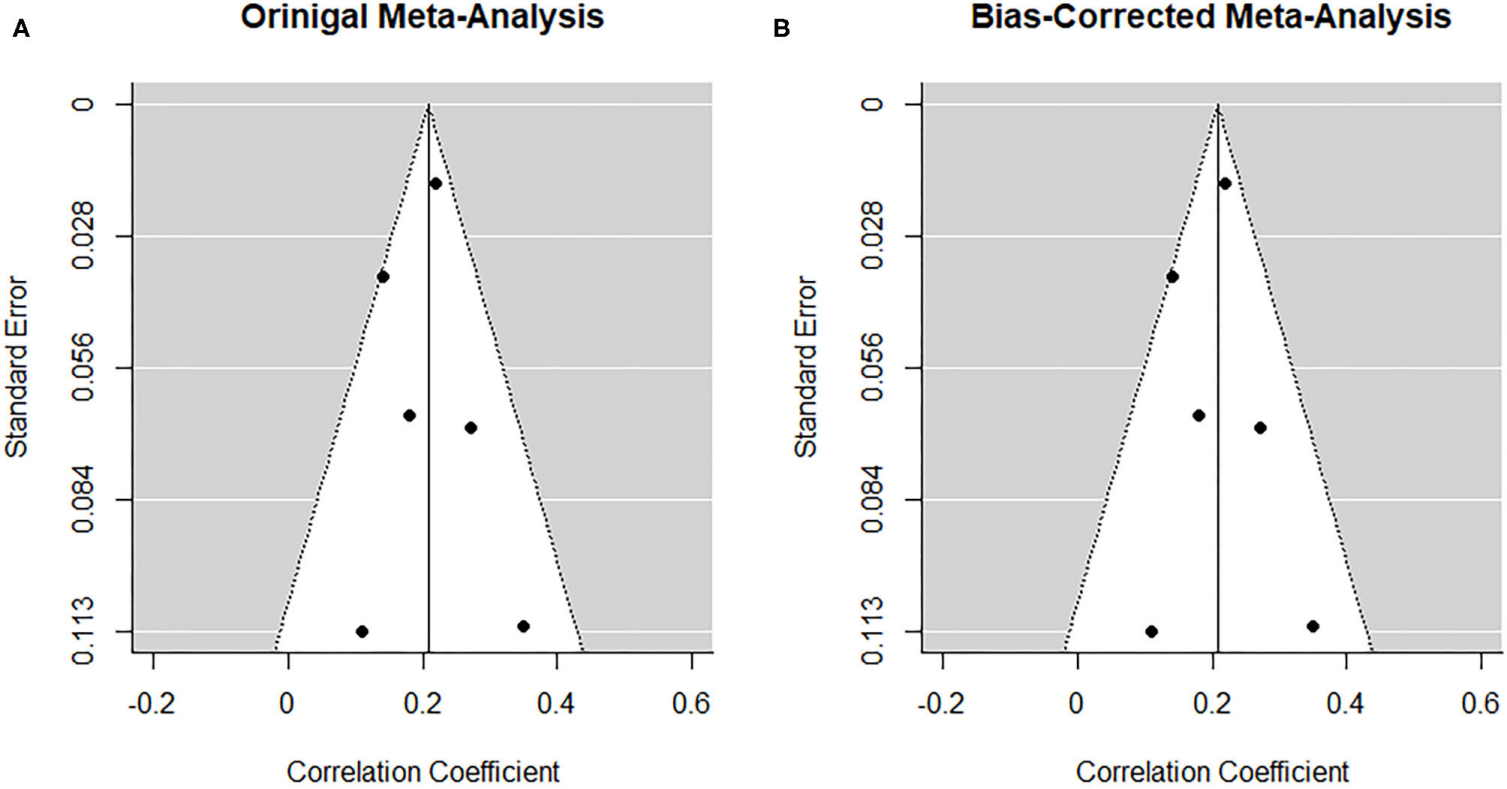
Funnel plots before **(A)** and after **(B)** applying the trim-and-fill method corresponding to the prospective association between maternal depression at one timepoint and paternal depression at a later timepoint during the perinatal period.

The low number of studies again limits interpretability of the following procedures. From visual inspection, the funnel plot (Figure 9A) appears symmetrical. In line with the visual inspection, the trim-and-fill procedure (34) lends further support (Figure 9B), and the regression test for funnel plot symmetry (33) was not statistically significant (*z* = 0.26, *p* = 0.793).

##### *Post hoc* analyses

Two of the 28 included studies (47, 65) reported correlations between paternal and maternal depressive symptoms using both the EPDS and the CES-D. As noted above, for the main analysis we included reported EPDS effects. Nonetheless, in the previous meta-analysis (10), depression measures were selected based on their development for both men and women (e.g., CESD) vs. their development for postpartum women (e.g., EPDS). We therefore followed the same steps described above to additionally run meta-analyses using the reported CES-D effects.

The overall pooled estimate for the association between paternal and maternal depression during pregnancy remained stable, *r* = 0.243 (95% CI [0.161, 0.325], *z* = 5.82, *p* < 0.0001), as did the overall pooled estimate for the prospective association between paternal and maternal depression during the perinatal period, *r* = 0.20 (95% CI [0.136, 0.264], *z* = 6.12, *p* < 0.0001). The overall pooled estimate increased slightly for the association between paternal and maternal depression during the postnatal period, *r* = 0.291 (95% CI [0.206, 0.375], *z* = 6.74, *p* < 0.0001) and the prospective association between maternal and paternal depression, *r* = 0.211 (95% CI [0.183, 0.240], *z* = 14.70, *p* < 0.0001).

##### Moderator analysis

Across studies, we observed substantial variability in postnatal assessment timepoints, ranging from within 2 days (59) to 9 months (56) following birth (Table 1C). To examine postnatal assessment timepoint as a moderator, we divided studies into three categories, based on time intervals commonly employed in the literature, namely, (a) within 1 month, (b) between 1 and 3 months, and (c) more than 3 months following birth. Postnatal assessment timepoint did not moderate the association between maternal and paternal depressive symptoms during the postnatal period [*Q*(2) = 1.12, *p* = 0.57].

## Discussion

In this systematic review and meta-analysis of studies published within the last decade, we aimed to expand current knowledge regarding the relationship between paternal and maternal depression in expectant and new fathers and mothers. Overall, four separate meta-analyses were performed. Despite considerable variability, pooled estimates provide overwhelming evidence for (1) a positive association between paternal and maternal depressive symptoms during pregnancy, (2) following birth, and (3) a prospective association between parental symptoms. Interpretation of the latter is limited because of a small number of studies reporting a prospective association.

Our findings add to the 2010 meta-analysis by Paulson and Bazemore (10), which synthesized 13 estimates pertaining to the correlation between paternal and maternal depressive symptoms in the perinatal period, reporting a pooled association of *r* = 0.308. Overall, the results reported herein are comparable to those of the previous meta-analysis (10). It should, however, be noted that Paulson and Bazemore's main focus was on the point prevalence of paternal depressive symptoms. In contrast, we focused on the relationship between maternal and paternal symptoms before and after birth, as well as on the prospective relationship between the two in both directions. When comparing the pooled effect sizes reported herein with those reported by Paulson and Bazemore, it should be taken into consideration that we conducted separate random-effects analyses for prenatal and postnatal timepoints, whereas Paulson and Bazemore included effects from both periods in one analysis. Taken together, however, the previous meta-analysis and our findings indicate a positive association between paternal and maternal depressive symptoms during pregnancy and following birth.

It must be noted that considerable variability was observed in reported relationships between paternal and maternal depression. This heterogeneity is indicative of between-study differences, although caution is warranted when interpreting the indices of heterogeneity in small meta-analyses (68). Nonetheless, closer inspection of the studies identified in our literature search revealed important between-study variation. For example, studies varied in the exact timepoint of assessment. Unfortunately, study count did not allow for inclusion of assessment time as a moderator in the meta-analyses regarding parental depression during pregnancy and the prospective association. We could, however, perform moderation analysis for the association between paternal and maternal depression during the postnatal period. Results indicated that the pooled estimate did not vary significantly depending on assessment within 1 month, between 1 and 3 months, or more than 3 months following birth.

The multitude of study locations may offer another possible explanation for the observed heterogeneity. The 28 studies included in the present meta-analyses stemmed from 16 different countries. Although this variability did not allow for meaningful moderator analysis, one could argue for the practical relevance of variability due to different locations, which in this case may offer valuable information for scientific practice. Different study locations may represent culturally diverse samples, particularly with regard to romantic relationship arrangements, as well as living/cohabiting, and work and parenting practices. For instance, in some regions of the world, fathers may be somewhat more involved in their partners' pregnancy and infant care, whereas in other regions, more conservative and traditional parenting roles may still prevail, ultimately also limiting fathers' time and involvement with mothers and newborns. Nonetheless, although pooled estimates were small to moderate in size, given the internationality of included effects and samples, we may conclude that the relationship between paternal and maternal depression in the perinatal period may be stable and observable across cultures.

Additionally, we observed that only one study utilized interview methods, all other studies relied on validated self-report measures to assess symptoms of depression. Thus, there were too few interview studies and too many different questionnaires to conduct reliable instrument-by-instrument moderator analyses. Further, the heavy reliance on subclinical samples consisting largely of individuals who may report depressive symptoms and impairment, but who do not meet full diagnostic criteria for depression (10, 69), should be taken into consideration in light of the so-called “postpartum blues.” The *postpartum blues* denotes deteriorations in mood during the first weeks following childbirth and affects up to 85% of mothers (70). How and if the *postpartum blues* manifests in fathers remain to be further investigated.

In the previous meta-analysis examining parental depression during the perinatal period, Paulson et al. (10) note that paternal depression has almost exclusively been examined in the context of studies focusing on index mothers or children. Over the past decade, this observation still seems to hold true. For instance, although marital/relationship dissatisfaction is among the strongest predictors of maternal depression (71), investigations of various relationship types and thus variability in parental couples are virtually nonexistent. To this end, examinations of parental depression in homosexual couples, adoptive parents, parents who do not cohabitate, or couples in which the male partner is not the biological father of the child can offer ample grounds for future scientific attention. In addition, the field of interpersonal partner violence has grown over the past years and may be a factor of interest to consider in future studies on perinatal parental depression.

Further, causal relationships between paternal and maternal depression have been proposed in the past (9, 72, 73), but none of the studies included in this meta-analysis offered methodology appropriate to establish causality. Although based on a small number of studies, our meta-analysis on the prospective relationship suggests that paternal depressive symptoms during the perinatal period can predict maternal depressive symptoms at a later point in time. Nonetheless, future investigations pertaining to the direction of effect are warranted in particular in order to assess means of screening and prevention.

It should additionally be acknowledged that several studies could not be included in this meta-analysis as a result of the statistical estimates reported. For instance, two studies were identified in which partners' depressive symptoms were implemented in multivariate regression models to predict maternal postnatal depression (35, 36). Both studies reported partners' symptoms to significantly predict maternal depression while taking into account various other predictors (35, 36). Two additional studies (37, 38) reporting congruence rates of symptom trajectories over the perinatal period could not be included in this meta-analysis. Whereas, Kiviruusu et al. (38) reported that paternal and maternal trajectories were highly associated with each other, Korja et al. (37) noted considerable congruence only between fathers and mothers categorized in the consistently low symptom trajectories. Considering the overwhelming reliance on community samples in which depressive symptoms were generally low, these studies further support the pooled estimates reported in this meta-analysis.

## Limitations

Several limitations of the current meta-analysis should be noted. First, because studies varied in methodology and assessment timepoints, considerable heterogeneity was observed. Although we utilized four separate random-effects meta-analyses to clarify associations at different timepoints during the perinatal period and were able to establish that assessment time was not a significant moderator of the association during the postnatal period, the rather small number of studies included may have overshadowed relevant time differences. Second, potential bias in our results may stem from limitations of the individual studies included herein. For instance, point estimates were drawn from a pool of studies heterogeneous in terms of study country, but rather homogeneous in terms of couple characteristics with the vast majority being married and/or cohabiting. Further, all but one study included relied solely on self-report measures, limiting the interpretation of our results to depressive symptoms in subclinical populations rather than couples with clinical diagnoses. Third, our findings suggest potential publication biases pertaining to studies included in the prenatal and postnatal meta-analyses. Interestingly, however, tests did not point toward missing null results, but rather, potential missing studies were identified as those reporting stronger effects. Given the unlikeliness of unpublished strong effects, we must take into account that all procedures utilized here perform only adequately in light of a limited number of studies and considerable heterogeneity (34, 74). Lastly, because gold-standard procedure for reviews includes data extraction by two independent raters, it should be acknowledged that study screening and data extraction were only performed by FT, although M-MP cross-checked extracted data.

## Conclusion

Keeping in mind the aforementioned limitations, this meta-analysis provides further evidence for a low to moderate association between paternal and maternal depressive symptoms during pregnancy, in the postnatal period, as well as prospectively. These findings offer several implications for clinical practice. In most countries, the perinatal period represents a time of rather frequent medical contacts. Positive screening of mothers should therefore prompt clinical attention to fathers. Similarly, preventive and clinical efforts should take into account the family system and partners in particular, rather than focusing solely on the individual. Although it is beyond the scope of this meta-analysis to address the interesting issue of how the co-occurrence of maternal and paternal depressive symptoms affects respective outcomes, given the notable heterogeneity and limitations in currently reported studies, as well as the increasing evidence for the implications of parental depression for emotional and behavioral child development (13–19), further scientific attention is certainly warranted.

Future research in this area should focus on inclusion of couples, in various constellations, and at-risk couples in order to shed further light onto the interdependence of depressive symptoms in couples during the transition to parenthood. Besides contributing to our growing understanding in the field, this may enhance early identification and offer grounds for improving preventive and clinical interventions in order to optimize perinatal mental health services, ultimately benefiting mothers, fathers, and children.

## Data Availability Statement

The original contributions presented in the study are included in the article/supplementary material, further inquiries can be directed to the corresponding author/s.

## Author Contributions

FT and SG-N designed and conceptualized the present study. FT conducted manuscript screening and data extraction and wrote the first draft of the manuscript. M-MP performed the statistical analysis. SG-N and H-UW supervised data extraction and drafting of the manuscript. All authors contributed to the manuscript revision and all read and approved the submitted version.

## Conflict of Interest

The authors declare that the research was conducted in the absence of any commercial or financial relationships that could be construed as a potential conflict of interest.
